# “Predictors of in-hospital mortality in adult cancer patients with COVID-19 infection presenting to the emergency department: A retrospective study”

**DOI:** 10.1371/journal.pone.0278898

**Published:** 2023-01-26

**Authors:** Imad El Majzoub, Nour Kalot, Malak Khalifeh, Natalie Estelly, Tharwat El Zahran

**Affiliations:** 1 Department of Emergency Medicine, American University of Beirut Medical Center, Beirut, Lebanon; 2 Faculty of Medicine, American University of Beirut, Beirut, Lebanon; Delta State University, NIGERIA

## Abstract

**Background:**

Adult cancer patients are at higher risk of morbidity and mortality following COVID-19 infection. Being on the front lines, it is crucial for emergency physicians to identify those who are at higher risk of mortality. The aim of our study was to determine the predictors of in-hospital mortality in COVID-19 positive cancer patients who present to the emergency department.

**Methods:**

This is a retrospective cohort study conducted on adult cancer patients who presented to the ED of the American university of Beirut medical center from February 21, 2020, till February 21, 2021, and were found to have COVID-19 infection. Relevant data was extracted and analyzed. The association between different variables and in-hospital mortality was tested using Student’s *t* test and Fisher’s exact test or Pearson’s Chi-square where appropriate. Logistic regression was applied to factors with p <0.2 in the univariate models.

**Results:**

The study included 89 distinct patients with an average age of 66 years (± 13.6). More than half of them were smokers (52.8%) and had received chemotherapy within 1 month of presentation (52.8%). About one third of the patients died (n = 31, 34.8%). Mortality was significantly higher in patients who had recently received chemotherapy (67.7% vs 44.8%, p = .039), a history of congestive heart failure (CHF)(p = .04), higher levels of CRP (p = 0.048) and/or PCT(p<0.04) or were tachypneic in the ED (P = 0.016).

**Conclusions:**

Adult cancer patients with COVID-19 infection are at higher risks of mortality if they presented with tachypnea, had a recent chemotherapy, history of CHF, high CRP, and high procalcitonin levels at presentation.

## Introduction

At the time we believed we were at the mountaintop of Medicine, China declared the emergence of the novel, highly contagious Severe Acute Respiratory Syndrome Coronavirus 2 (SARS-CoV- 2). Coronavirus disease 2019 (COVID-19) was first detected in Wuhan, China [1]. In no time, SARS-CoV- 2 found its way to the whole world, to be declared as a pandemic by the World Health Organization (WHO) on March 11th, 2020. Its rapid spread and repercussions affected the world’s social, economic, and particularly medical sectors [2].

As this virus continues to take its toll on the world, it showed an ability to cause severe disease among certain groups, including older populations and individuals with underlying health conditions such as cancer [3]. Despite the debate about the strength of evidence, the literature suggests that cancer patients are more susceptible to COVID-19 infection compared to the general population [4]. Although COVID-19 does not seem to affect all cancer patients equally, these patients were generally found to be at higher risk of developing severe COVID-19 illness and dying from the infection [4]. Mehta et al found that COVID-19 infection could increase the risk of mortality regardless of the cancer type [5]. In a nationwide cohort study in China, Liang et al. found that the patients with cancer not only had a higher risk of SARS-CoV-2 infection, but also displayed an increased risk of severe clinical events (admission to the intensive care unit, need for invasive ventilation, or death) than those without cancer [6]. In their meta-analysis involving 23736 cancer patients, Venkatesulu et al. showed that cancer patients with COVID-19 have an increased likelihood of death compared with noncancer COVID-19 patients [7]. Data from the COVID-19 and Cancer Consortium (CCC19) cohort study, which included 1,018 patients, also reported that mortality and severe illness in COVID-19 cancer patients were significantly higher than in the general population [8].

Since the beginning of the COVID-19 outbreak in most countries, Emergency departments (EDs) have been on the frontline serving an essential role in identifying likely patients with the infection and isolating them early [9]. When it comes to cancer patients with COVID-19, their disposition from the ED is a tough decision to take [10]. On one hand, this owes to the life-threatening nature of cancer and the immense burden that the novel virus has placed on the already strained healthcare system [11–13]. On the other hand, the decision is made more complicated due to the significant amount of uncertainty regarding the influence of cancer on the outcomes of the infection [14].

It is therefore crucial that we study the variables that may be associated with a higher likelihood of in-hospital mortality in cancer patients presenting to the ED with COVID-19 infection to provide optimal care and prevent the grave consequences they are at risk of. Furthermore, identifying such variables may potentially help develop a predictive tool to identify COVID19-positive cancer patients with a higher risk of mortality.

## Methods

### Study design

This retrospective cohort study was approved by the institutional review board (IRB) under the protocol number (BIO-2021-0015). Informed consent was waived given the retrospective nature of the study.

The study was conducted on adult cancer patients who tested positive for Covid-19 infection upon presentation to the emergency department (ED) of the American University of Beirut Medical Center (AUBMC), between February 21, 2020, and February 21, 2021.

The AUBMC is the largest tertiary care and academic center in Lebanon. The center operates 376 beds and receives approximately 55,000 Emergency Department (ED) visits and approximately 25,000 inpatient admissions annually. The emergency department (ED) of AUBMC has 43 beds and has an onsite coverage of emergency physicians 24-hr/7.

### Study population

All adult (>18 years old) cancer patients who presented to the ED of AUBMC from February 21, 2020, till February 21, 2021, and were COVID-19 positive at presentation, were eligible for inclusion.

COVID-19 is defined as a positive result of SARS-COV-2 nucleic acid RT-PCR test using the nasal swab samples. Patients who presented dead to the ED were excluded.

### Data collection and sampling

All adult cancer patients presenting to the ED with COVID-19 infection were identified through the electronic health system (Epic Systems, Verona, WI, USA). In order to protect patients’ information and confidentiality, subject names were not collected. Each patient was anonymously assigned a study ID in the data collection sheet. The patient’s study ID were kept on a separate log sheet and were only accessible by the primary investigator and the research coordinator.

All patient information were entered by the postdoctoral research assistants, into REDCap, a free, secure, web-based application designed to support data capture for research studies that is Health Insurance Portability and Accountability Act compliant. The data included different sections. The first section included patient demographics, history of smoking (former or current smoker). The second part included cancer history and treatments, medical comorbidities, vitals at triage, ED management, length of ED stay, requirement of ICU admission, laboratory panel (included CRP, Procalcitonin, Troponin T and D-dimer), and disposition status. The data collection sheet is attached as appendix.

### Statistical analysis

Statistical analysis was performed using SPSS version 25.0 (Armonk, NY: IBM Corp). Categorical variables were described using frequencies and percentages. Continuous variables were reported using means, standard deviations. The variables were compared between patients who died to those who survived. The variable difference was calculated using Pearson’s Chi-square or Fisher’s exact test and Student’s *t* test where appropriate.

If a variable had missing data, percentages were calculated as per the remaining number of available data points. A p-value < 0.05 was considered significant. A logistic regression was then performed to determine the risk factors associated with mortality. The following variables were entered in the logistic regression: Vasopressors, Tocilizumab, Treatment with steroids in ED, Anticoagulants in ED, C-reactive protein, procalcitonin, Chemotherapy within 1month of presentation, respiratory rate at triage (reference: RR<22), O_2_ triage (reference: O_2_ saturation >95mmHg). We selected them based on a p-value > 0.2

## Results

### Demographics and clinical characteristics of COVID-19 oncology patients

As shown in Table 1, a total of 89 oncology COVID-19 patients were included in the study. Their average age was 66 years (± 13.6). Most patients were males (64%) and with solid cancer (73.3%). More than half of the patients with solid tumors had metastasis (52.3%). Hypertension was the main comorbidity among patients (39.3%), followed by cardiovascular diseases (25.8%), dyslipidemia (23.6%), and diabetes mellitus (14.6%). About half of them were smokers (52.8%) and had received chemotherapy within 1 month of ED presentation (52.8%). Only 6 patients did BMT within 1 year of presentation. About third of the patients died (n = 31, 34.8%) were admitted to the ICU (n = 33, 37%) (Table 1).

**Table 1:** Association of baseline characteristics of oncology COVID-19 patients with in-hospital mortality.

Characteristics		Total N = 89	Alive N = 58	Dead N = 31	p value	OR	95%CI
**Age (years)**		66.3 (13.6)	65 (13.8)	68.7 (12.9)	0.233		
**Sex**	**Female**	32 (36%)	21 (36.2%)	11 (35.5%)	0.946	Reference	
	**Male**	57 (64%)	37 (63.8%)	20 (64.5%)		1.032	0.415–2.564
**History of smoking**		47 (52.8%)	30 (51.7%)	17 (54.8%)	0.779	1.133	0.472–2.719
**Type of Cancer**	**Liquid**	23 (26.7%)	16 (28.6%)	7 (23.3%)	0.601	Reference	
	**Solid**	63 (73.3%)	40 (71.4%)	23 (76.7%)		1.314	0.471–3.665
**Metastatic**		34 (52.3%)	22 (52.4%)	12 (52.2%)	0.987	0.992	0.358–2.744
**Bone Marrow Transplant within 1 year**		6 (6.8%)	3 (5.3%)	3 (9.7%)	0.661	1.929	0.365–10.185
**Chemotherapy within 1 month**		47 (52.8%)	26 (44.8%)	21 (67.7%)	0.039	2.585	1.037–6.445
**Immunotherapy**		19 (21.3%)	12 (20.7%)	7 (22.6%)	0.836	1.118	0.389–3.21
**Comorbidities**	**Cardiovascular Diseases**	23 (25.8%)	9 (21.4%)	14 (29.8%)	0.369	1.556	0.592–4.089
	**Congestive heart failure**	3 (3.4%)	0 (0%)	3 (9.7%)	0.04		
	**coronary artery diseases/ arrhythmias**	22 (24.7%)	12 (20.7%)	10 (32.3%)	0.228	1.825	0.682–4.889
	**Diabetes Mellitus**	13 (14.6%)	9 (15.5%)	4 (12.9%)	1	0.807	0.227–2.866
	**Hypertension**	35 (39.3%)	25 (43.1%)	10 (32.3%)	0.318	0.629	0.252–1.569
	**Dyslipidemia**	21 (23.6%)	15 (25.9%)	6 (19.4%)	0.491	0.688	0.237–2.001
	**Dementia**	1 (1.1%)	0 (0%)	1 (3.2%)	0.348		
	**Cerebrovascular accident/Transient ischemic attack**	2 (2.2%)	1 (1.7%)	1 (3.2%)	1	1.9	0.115–31.459
	**Chronic Obstructive Pulmonary Disease**	8 (9%)	6 (10.3%)	2 (6.5%)	0.708	0.598	0.113–3.155
	**Chronic Kidney Disease**	16 (18%)	9 (15.5%)	7 (22.6%)	0.408	1.588	0.528–4.779
	**Hemiplegia**	1 (1.1%)	1 (1.7%)	0 (0%)	1		
	**Peptic ulcer disease**	2 (2.2%)	1 (1.7%)	1 (3.2%)	1	1.9	0.115–31.459
	**Liver Disease**	3 (3.4%)	1 (1.7%)	2 (6.5%)	0.277	3.931	0.342–45.179
	Other*	58 (65.2%)	39 (67.2%)	19 (61.3%)	0.575	0.771	0.311–1.911

Data are presented as numbers with percentages. P-value for difference between two adjacent columns is calculated by chi-square or Fisher´s exact test where appropriate.

Abbreviations: OR: odds ratio, 95%CI: 95% Confidence Interval.

*Other comorbidities are thyroid disease, psychiatric disorders, and rheumatologic diseases.

For the vital signs, about 40.4% had low oxygen saturation at triage < 95% (n = 36, 40.4%) and most of the patients had tachycardia (n = 43, 48.3%) (Table 2).

**Table 2:** Association of vital signs and ED treatment of COVID oncology patients with in-hospital mortality.

		Total	Alive	Dead	p value	OR	95%CI
		N = 89	N = 58	N = 31			
ED treatment							
**Mechanical Ventilation in ED**		11 (12.4%)	4 (6.9%)	7 (22.6%)	0.044	3.938	1.053–14.728
**Blood Transfusion**		11 (12.4%)	4 (6.9%)	7 (22.6%)	0.044	3.938	1.053–14.728
**Platelet transfusion**		6 (6.7%)	1 (1.7%)	5 (16.1%)	0.018	10.962	1.219–98.589
**Vasopressors**		7 (7.9%)	2 (3.4%)	5 (16.1%)	0.047	5.385	0.979–29.608
**Steroid**		50 (56.2%)	27 (46.6%)	23 (74.2%)	0.012	3.301	1.269–8.584
**Actemra**		8 (9%)	2 (3.4%)	6 (19.4%)	0.02	6.72	1.267–35.637
**Baricitinib**		3 (3.4%)	0	3 (9.7%)	0.04		
**Vital Signs**							
**Respiratory rate at triage**	**< = 22**	72 (83.7%)	51 (91.1%)	21 (70%)	0.016	Reference	
	**>22**	14 (16.3%)	5 (8.9%)	9 (30%)		4.371	1.309–14.595

Data are presented as numbers with percentages.

P-value for difference between two adjacent columns is calculated by chi-square or Fisher´s exact test where appropriate.

Abbreviations: OR: odds ratio, 95%CI: 95% Confidence Interval, Ref = Reference, ED = emergency department

### Treatments and health-related complications of COVID oncology patients

In the emergency department, COVID-19 oncology patients were treated with steroids (56.2%), antibiotics (48.3%), and anticoagulants (47.2%). They were also treated with Remdesivir (19.1%), Ivermectin (14.6%), Tocilizumab (9%), or convalescent plasma (6.7%). Only 7 patients were treated with vasopressors (7.9%) (Table 2).

Moreover, 33.7% developed respiratory complications including ARDS, pneumothorax, or respiratory failure while 15.7% had septic shock and 7.9% developed cardiovascular complications. Only 8 patients required dialysis (9%) and 28.1% required endotracheal intubated (n = 25). The average length of hospital stay was 30.7 days (+- 65.1) (Table 2).

None of the comorbidities, age, or smoking status were significantly different between patients with liquid or solid tumors (p > 0.05). However, the majority of patients with liquid tumors were males (95.7% vs 50.8%, p<0.001) and had more moderate to severe kidney diseases (34.8% vs 11.1%, p = .021).

### Predictors of in-hospital mortality in COVID-19 cancer patients

There was no significant difference in gender, age, smoking status, and presence of comorbidities between COVID-19 oncology patients who died versus those who did not die. Of the total 31 patients who died, the average age was 68.7 years (± 12.9) and 64.5% were males (n = 20). Mortality was significantly higher in patients who had received chemotherapy within 1 month of presentation to the ED (67.7% vs 44.8%, p = .039) and in patients who had a history of CHF (p = 0.04) (Table 1).

For the vital signs, patients with tachypnea at triage (>22) were 4 times more associated with mortality (30% vs 8.9%, p = .016). There was no significant difference in systolic blood pressure, oxygen saturation and temperature of patients in ICU compared to general admission (p > 0.05) (Table 2).

Patients who died were significantly more admitted to ICU (74.2% vs 17.2%, p<0.001). They were significantly more treated with vasopressors (16.1% vs 3.4%, p<0.001) and more mechanically ventilated (22.6% vs 6.9%, p = .044) in the ED. They were also significantly more treated with blood transfusion (22.6% vs 6.9%, p = 0.044) and with platelet transfusion (16.1% vs 1.7%, p = 0.018) (Table 2).

In the ED, patients who died were also significantly more on Barictinib (p = .04) and 6.7 times more on Tocilizumab (19.4% vs 3.4%, p = .02) and 3.3 times more on steroids (74.2% vs 46.6%, p = .012). Compared to those who survived, patients who died had significantly elevated CRP level (125.7±97.8 vs 86.2±74.4, p = 0.048) and procalcitonin levels (2.2±4.6 vs 0.2 ± 0.2±0.2, p < 0.044) (Table 3).

**Table 3:** Association of laboratory data of COVID-19 oncology patients at presentation to the emergency department with in-hospital mortality.

Laboratory Data	Total N = 89	Alive (N = 58)	Dead (N = 31)	P value
**White blood cells count**	8735.830 (11719.0215)	7288.2 (7981.99)	11397.5 (16371.97)	0.196
**Hemoglobin**	11 (1.9456)	11.2 (1.93)	10.7 (1.97)	0.316
**Platelets**	184323.864 (92905.1327)	178635.1 (89323.58)	194783.9 (99813.99)	0.439
**Lactate Dehydrogenase**	568.77 (560.581)	494.9 (397.62)	681.8 (745.26)	0.29
**Lactic acid Venous**	1.9024 (1.39752)	1.6 (0.5972)	2.4 (2.02)	0.128
**C-Reactive Protein**	99.5 (84.5)	86.2 (74.4)	125.7 (97.8)	0.048
**d-dimer**	1379.9 (3418.1)	938.5 (1063.7)	2205.1 (5591.6)	0.293
**Procalcitonin**	1 (2.9)	0.2 (0.2)	2.2 (4.6)	0.044
**Troponin T**	0 (0.1)	0 (0)	0 (0.1)	0.25

Data are presented as mean with standard deviation.

P-value for difference between two adjacent columns is calculated by T test.

The frequency of complications was significantly higher in patients who died compared to those who survived significantly as follows: respiratory complications (77.4%, p < .001), AKI (p < .001) and septic shock (p < .001). They were significantly more on Dialysis (p = 0.02) (Table 2).

### Predictors of mortality using logistic regression

We found the increase in PCT levels and respiratory rate at triage to be associated with increased risk of mortality (aOR = 5.899, 95%CI = 1.2–29.3; aOR = 6.95, 95%CI = 1.3–36.9 respectively). Moreover, patients who died were more likely to receive Tocilizumab in our ED (aOR = 8.368, 95%CI: 1.27–55.127) (Table 4).

**Table 4:** Logistic regression: Factors associated with in-hospital mortality in COVID-19 oncology patients.

	p-value	aOR	95% C.I.	
Tocilizumab	0.027	8.368	1.27	55.127
Procalcitonin	0.03	5.899	1.189	29.252
RR at triage	0.023	6.947	1.309	36.854

Variables entered in model: Vasopressors, Tocilizumab, Treatment with steroids in ED, Anticoagulants in ED, C-reactive protein, procalcitonin, Chemotherapy within 1month of presentation, respiratory rate at triage (reference: RR<22), O_2_ triage (reference: O_2_ saturation >95mmHg).

Omnibus <0.001, R2 = 0.549, Hosmer = 0.672.

95%C.I.: 95% Confidence Interval, aOR: adjusted Odds Ratio.

## Discussion

Based on our observations, a physician should be alarmed if the COVID-19 positive cancer patient presenting to the ED has a history of CHF, received chemotherapy within 1 month, is tachypneic (with respiratory rate above 22), or if his initial lab workup showed an elevated C-Reactive protein and/or procalcitonin values.

In contrast to many studies [15, 16], age was not found to be significantly associated with mortality in our cohort. This was justified by Zhao et al, who considered that comorbidities related to aging, rather than advanced age itself, are what contribute to a worse prognosis [17]. Other issues of controversy between our results and the current data include male sex and hematologic malignancies which were both shown to be top predictors of mortality in various studies. These differences may be explained by the discrepancies in the population’s baseline characteristics or the choice of the statistical analyses.

The only comorbidity that stood out as a significant predictor for mortality in the univariate analysis of this study was heart failure. This has also been reported in other studies. Patients with cardiovascular disease, namely, heart failure were found to be more susceptible to coronavirus disease 2019 (COVID-19) and have a more severe clinical course once infected [18]. In their meta-analysis of 20 individual studies Dalia et al. explained this by the fact that COVID-19 can cause hypoxia induced excessive intracellular calcium, and since CHF patients are recognized to have dysregulation of intracellular calcium handling mechanism, the combination of COVID-19 and CHF in a patient leads to cardiac myocyte apoptosis [19].

Whether chemotherapy is a predictor of mortality in cancer patients with COVID-19 infection is still debatable. Several published articles haven’t found a relation between cancer treatment and mortality [20–22]. However, there are several others which proposed that chemotherapy plays a role in increasing COVID-19 death rates. In a study that included 205 COVID-19 cancer patients Yang et al found that chemotherapy within 4 weeks before COVID-19 symptom onset is a risk factor for death [23]. Similarly, in a follow-up analysis of the COVID-19 and Cancer Consortium study that included more than 4000 patients, anticancer therapies, particularly chemotherapy and antilymphocyte therapy, were found to be associated with high 30-day mortality [24]. Such results were also consistent with those of the large National COVID-19 Cohort Collaborative, which found that chemotherapy within 30 days was associated with an increased mortality risk [25]. In this context, if we were to comment on the findings of our studies, the multivariable analysis showed that receiving chemotherapy imposes no greater risk of death during infection with COVID-19. This reflects the importance of other variables and indicates the necessity of conducting large multicentered studies.

Concerning predictors of respiratory compromise, we found that a respiratory rate exceeding 22 breaths per minute at initial presentation, is associated with elevated mortality rates in hospitalized cancer COVID-19 patients. In fact, it was also shown to increase the risk of severe outcomes in COVID-19 [26]. Chatterjee et al reported that compared to patients with normal respiratory rate (≤20 beats per minute), those with respiratory rates more than 22 breaths per minute, had 1.9 to 3.2 fold elevated mortality risk [27]. A Chinese study on 344 critically ill patients also found higher respiratory rate to be associated with poor outcome indicating that more attention need to be paid to vital signs [28]. All these observations should not be surprising given the fact that respiratory rate is an integral component of many severity scoring systems like CURB65 (Confusion, Urea, Respiratory rate, Blood pressure, Age > 65 years) score and PSI (Pneumonia Severity Index) [29–31]. Indeed, this is an additional testimony on the importance of respiratory rate and its monitoring.

As for serology, we found that higher C-reactive protein (CRP) levels were associated with an increased risk of mortality. Earlier reports have also indicated that higher values of CRP during the initial stages of the disease were associated with greater CT severity scores and extensive lung involvement [32]. CRP which is an acute-phase reactant that is synthesized by the liver in the setting of infection or tissue injury plays an important role in producing proinflammatory cytokines [33–35]. One of the key findings of immunopathology in COVID-19 is the cytokine storm [36]. As the virus rapidly replicates in the host’s cells, this triggers the immune system to develop significant numbers of proinflammatory cytokines and chemokines [37]. The development of larger quantities of proinflammatory cytokines implies a more severe COVID-19 infection which can eventually lead to complications such as acute respiratory distress syndrome (ARDS) and multiple organ failure, and ultimately higher mortality in patients with COVID-19 [38].

From this dataset, using multivariate analysis, we concluded that procalcitonin (PCT) can be considered as one of the top predictors of mortality. While under normal conditions, PCT is produced exclusively by the thyroid gland, when challenged with infection, a significant production of PCT by nonthyroidal tissues occurs [35]. The release of PCT is mediated by proinflammatory cytokines, such as tumor necrosis factor-alpha and interleukin-6 [39]. Procalcitonin is widely considered to be the useful marker of severe systemic inflammation because of its valuable role in the diagnosis and prognosis in regards to sepsis [40]. Compared to CRP, PCT level rises rapidly and pikes within very short time frame [35]. While it is still uncertain whether PCT can accurately distinguish bacterial or viral pneumonia [41], in the case of COVID-19, elevated levels of PCT were associated with a five-fold greater risk of severe disease progression [42]. Furthermore, in a meta-analysis investigating the association between inflammatory markers and prognosis in COVID-19 patients higher procalcitonin levels were associated with increased [43]. Some contributed this to secondary bacterial infections which are common in COVID-19 pneumonia especially that it was noted that serum PCT concentrations stay within the normal range in uncomplicated COVID-19 cases and inflated values may indicate bacterial co-infection in severe cases [42].

It should be noted that in our ED, Tocilizumab was given to sicker patients. This would explain the high risk of mortality in these patients seen in Table 3. Scarce information is available regarding the use of this recombinant humanized monoclonal antibody in cancer patients with COVID-19 infection [44]. And early data which suggested its efficacy in treating COVID-19 is limited to observational studies and case reports [44, 45]. In order to have valid answers regarding this drug, randomized clinical trials with large sample sizes are needed [46].

### Limitations

The retrospective nature of our study, its small sample size, and the fact that it was done in a single institution, all are important shortcomings of our study. Furthermore, owing to the evolving nature of the virus, the advancement of treatment strategies as well as the vaccine rollout, the results might be affected. We should also mention that we were not able to analyze various factors such as the patients’ BMI and laboratory values including ferritin and others because data on these variables were missing in many patients. Combining these limitations, it is better to categorize our study as a descriptive one that gives hints to physicians rather than providing a concrete predictive tool.

## Conclusion

Based on our observations, when encountering a covid-19 positive cancer patient in the ED with history of CHF, with an elevated CRP and/or PCT levels, has received chemotherapy within 1 month, and/ or is tachypneic (respiratory rate >22), one can objectively predict an increased risk of mortality. Our findings would enable frontline health care workers to enhance the risk stratification of patients and guide the decision-making process in busy ED units. This serves the goal of delivering the best medical care to such a highly vulnerable group of patients. Further prospective studies are required to address the limitations this research had and ascertain the validity of the results.

## Supporting information

S1 File (XLSX)
